# Extradural malignant melanotic nerve sheath tumor of the lumbosacral spine: a diagnostic and surgical challenge

**DOI:** 10.1097/MS9.0000000000003327

**Published:** 2025-05-12

**Authors:** Naeem Ul Haq, Gulmeena Aziz Khan, Shehryar Haider, Ashan Fareed, Samim Noori, Sardar Noman Qayyum

**Affiliations:** a Department of Neurosurgery, Bacha Khan Medical College, Mardan, Pakistan; b Department of Neurosurgery, Lady Reading Hospital, Peshawar, Pakistan; c Department of Neurosurgery, Al-Nafees Medical College, Islamabad, Pakistan; d Department of Neurosurgery, Faculty of Medicine, Nangarhar University, Nangarhar, Afghanistan

**Keywords:** extradural spinal tumor, lumbosacral compression, malignant melanotic nerve sheath tumor, melanotic schwannoma

## Abstract

**Introduction and importance:**

Malignant melanotic nerve sheath tumor (MMNST) is a rare and potentially aggressive variant of schwannoma that arises from Schwann cells containing melanin. These tumors most frequently occur in the spinal nerve roots and are often misdiagnosed due to their radiological and histopathological similarities with other pigmented lesions such as malignant melanoma or pigmented meningioma. Accurate diagnosis is crucial, as MMNST carries a higher risk of local recurrence and distant metastasis compared to conventional nerve sheath tumors.

**Case presentation:**

We report the case of a 30-year-old male who presented with a 6-month history of progressive left-sided leg pain, lower backache, and paresthesia. Neurological examination revealed decreased perineal sensations and tenderness in the lumbosacral region. MRI of the lumbosacral spine showed an extradural, well-circumscribed, T1 hyperintense lesion with mass effect on the left L5, S1, and S2 nerve roots. The patient underwent surgical resection, and the lesion was found to be encapsulated and pigmented. Histopathology confirmed the diagnosis of MMNST. Immunohistochemistry was positive for S-100 and HMB-45. Postoperative recovery was uneventful, and no recurrence was observed at 6-month follow-up.

**Clinical discussion:**

MMNST poses significant diagnostic and therapeutic challenges. While MRI may suggest a pigmented lesion, definitive diagnosis relies on histopathology and immunohistochemical markers. Given its malignant potential, complete surgical resection with long-term follow-up is the mainstay of management. In this case, the tumor’s extradural location and involvement of the sacral nerve roots added to the surgical complexity. Multidisciplinary collaboration was pivotal in planning surgical excision and monitoring for recurrence.

**Conclusion:**

MMNST should be considered in the differential diagnosis of pigmented spinal lesions, especially in young adults with progressive neurological symptoms. Early recognition and complete surgical resection remain essential for optimal outcomes. This case highlights the importance of integrating imaging, histopathology, and immunohistochemistry to ensure accurate diagnosis and guide management.

## Introduction

Malignant melanotic nerve sheath tumor (MMNST), previously known as melanocytic schwannoma, is an exceedingly rare, aggressive neoplasm derived from melanin-producing Schwann cells of neural crest origin^[^1^]^. It exhibits spindle-shaped cells arranged in fascicles or nests, with melanin pigmentation. First described by W. Gilbert Miller in 1932 as a malignant melanotic tumor of ganglion cells,^[^1^]^ and was later named as melanocytic Schwannoma by Folpe *et al* in 1975^[^2^]^. MS, previously classified as benign in the 2013 WHO classification, was renamed as “Malignant Melanotic Nerve Sheath Tumor” in the 2020 WHO classification and was reclassified as malignant due to its unpredictable behavior, i.e., local recurrence and metastasis even in the absence of malignant histological features. MMNST comprises less than 1% of all nerve sheath tumors^[^3^]^ and typically presents as a localized mass, with symptoms due to compression of adjacent neurological structures such as pain, frailty, paresthesia, and motor dysfunction^[^4^]^. It can also occur along cranial nerves, but it most frequently occurs along the spinal nerve roots near the midline. Spinal MMNST mostly involves the lumbosacral region (47.2%), the thoracic region (30.5%), and the cervical region (22.2%)^[^4^]^. MRI/CT scans are performed for diagnosis, later confirmed by histopathology report. There is no curative treatment for MMNST^[^5^]^, surgical resection is performed which relieves the pressure symptoms along with adjuvant radiotherapy in metastatic spread^[^2^]^.

**HIGHLIGHTS**

- Malignant melanotic nerve sheath tumor (MMNST) is an exceptionally rare neoplasm, especially in the sacral extradural region, with fewer than 100 cases reported globally.

- This case involved a young adult male presenting with nonspecific lumbosacral symptoms, underlining the diagnostic challenges due to overlapping clinical features with common spinal pathologies.
- Histopathology and immunohistochemistry played a pivotal role in definitive diagnosis, distinguishing MMNST from other pigmented or nerve sheath tumors.
- Extensive metastatic work-up including contrast-enhanced computed tomography (CECT), MRI brain, and bone scintigraphy ruled out systemic spread, emphasizing the need for thorough evaluation in all cases.
- Complete surgical excision remains the mainstay of treatment, although long-term monitoring is essential due to MMNST’s unpredictable course and risk of recurrence.

In this article, we report the case of a 30-year-old male patient presenting with a history of progressive left-leg pain, backache, and paresthesia below the lumbosacral junction from the last 6 months. The patient was diagnosed via MRI with MMNST and the tumor was surgically resected, alleviating the patient’s symptoms. This article contributes to the existing literature by addressing the management and long-term follow-up of MMNST. We followed the SCARE 2023 guidelines to report this case^[^6^]^.

## Patient presentation

A 30-year-old male patient presented to OPD (Outpatient Department) with complaints of progressive left leg pain, paresthesia, and backache from the last 6 months. The symptoms were gradual in onset without any history of trauma. The patient had no family history of malignancy. His past medical and surgical history was unremarkable. Upon examination, the patient had tenderness at the lumbosacral junction along with decreased perineal sensations, while sphincter functions and motor functions were intact. On general physical examination, there were signs of anemia (pallor) for which baseline investigations were ordered.

### Investigations

**Figure 1.**
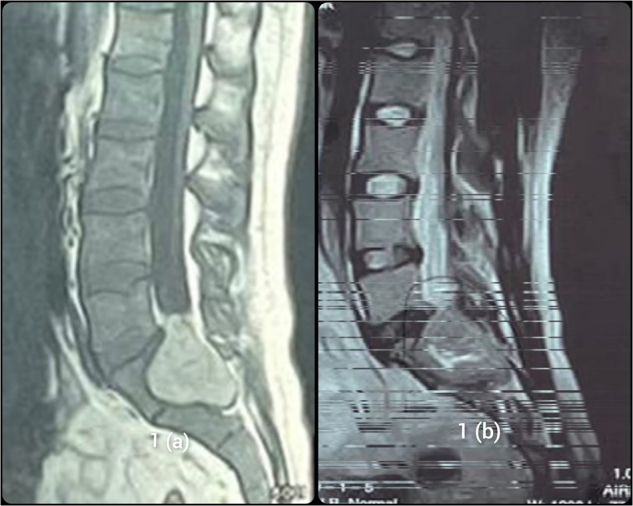
(a) and (b) show the sagittal sections of T1- and T2-weighted images, respectively.

Baseline investigations revealed anemia (Hb <8 mg/dl). An MRI scan was performed which revealed a tumor predominantly on the left side, compressing S1 and S2 nerve roots along with L5 lower segment, with more significant involvement on the left side and slight extension toward the right. The tumor was approaching the L5 vertebral body. Figure 1(a) and (b) depicts the sagittal sections of T1- and T2-weighted images (T1WI and T2WI), showing a bulky tumor at the lumbosacral junction, primarily compressing S1 and S2 nerve roots.

**Figure 2.**
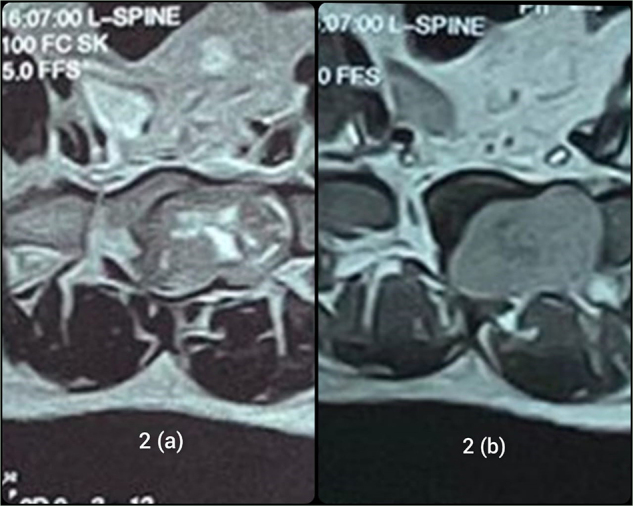
(a) and (b) show the axial views of T2- image and T1-weighted image, respectively.

Figure 2(a) and (b) provides the axial views of T2WI and T1WI, further illustrating the tumor compressing S1 and S2 regions.

### Differential diagnoses

The differential diagnosis included sacral chordoma, schwannoma, metastatic melanoma, and MMNST. The diagnosis of metastatic melanoma was excluded due to the absence of an identifiable primary lesion. The definitive diagnosis required histological and immunohistochemistry examination of the tumor after excisional biopsy.

### Treatment

The patient was counseled for the surgical resection of the tumor. Following the patient’s consent, the patient was optimized before surgery by anemic correction with three packs of whole blood. The surgical approach was aimed at full tumor resection to decompress the S1 and S2 nerve roots for alleviating the patient’s symptoms.

The surgical procedure was performed under general anesthesia. Following the midline incision on the lumbosacral region, posterior laminectomy was performed to create a window in the laminae of the affected vertebrae. This was followed by durotomy because part of the tumor had infiltrated the dura mater, compressing S1 and S2 nerve roots. Following the microsurgical excision, the specimen was sent for histopathological examination. Supplementary Video 1, http://links.lww.com/MS9/A809, shows the tumor before micro-surgical resection, while Supplementary Video 2, http://links.lww.com/MS9/A810, shows the space occupied by the tumor after surgical resection.

### Histopathological and immunohistochemistry findings

**Figure 3.**
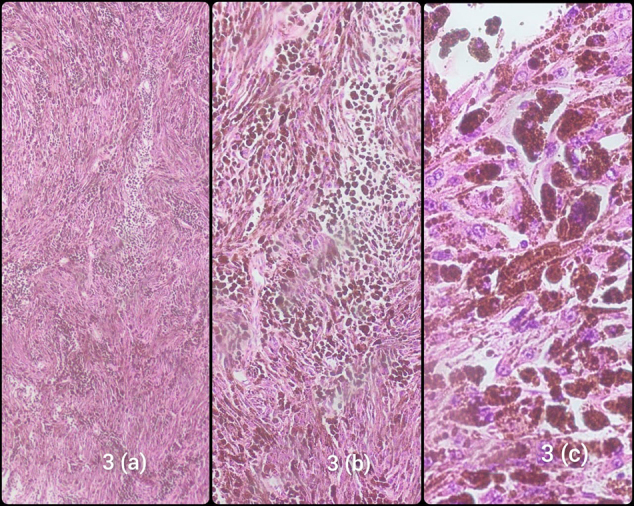
(a), (b), and (c) show the histopathology images of the specimen at 2×, 10×, and 20×, respectively.

The tumor was resected in multiple fragments, collectively measuring 45 mm × 35 mm × 15 mm on gross examination. Microscopic examination revealed short fascicles of spindle to ovoid cells arranged in a whirling pattern, with eosinophilic cytoplasm and round to oval nuclei. Abundant melanin pigmentation was also observed. Figure 3(a)–(c) contains the histopathology images of the specimen.

Immunohistochemistry revealed positive staining for the neural crest markers, namely HMB45 and SOX10. The histopathological and immunohistochemistry findings were consistent with the diagnosis of MMNST.

### Follow-up

The surgical team was cautious throughout the surgery due to the tumor’s location which posed a risk of compromising lower motor function. The patient was mobile, pain free, and with minimal complaints within 1 week. Due to unpredictable metastatic pattern and higher recurrences, he was referred to an oncologist for further follow-up. However, after 1 year of follow-up, the patient’s metastatic work-up was negative with no recurrence.

Table 1 shows the timeline of the events in this case.

**Table 1:** Timeline of clinical events

Timeframe	Event
Six months before visit	Patient began experiencing left leg pain, numbness, and intermittent backache.
Four months before visit	Symptoms progressively worsened; difficulty in prolonged walking and standing noted.
One month before visit	Pain became more persistent, associated with tingling and perineal discomfort.
Day 0 (hospital visit)	Patient presented to the outpatient department with worsening symptoms. Neurological examination revealed lower back tenderness and decreased perineal sensation.
Day 3	MRI of the lumbosacral spine showed an extradural mass compressing S1 and S2 nerve roots on the left side.
Day 5	Multidisciplinary team meeting held; surgical intervention planned.
Day 7	Surgical excision performed. Tumor appeared blackish with a bluish capsule; subtotal resection achieved.
Post-op Day 3	Histopathology confirmed diagnosis of *malignant melanotic nerve sheath tumor*.
Post-op Week 1	Patient discharged in stable condition; advised follow-up with oncology.
Post-op Month 1	Metastatic work-up (CT scan chest, abdomen, and pelvis) performed; no evidence of metastasis.
Follow-up Month 3	Neurological status stable; no new symptoms; MRI not done due to financial constraints.
Follow-up Month 6	Patient clinically stable; no recurrence or deterioration noted.
Follow-up Month 12	Final follow-up; patient asymptomatic, no radiological or clinical evidence of recurrence. No adjuvant therapy advised by oncology team.

### Take home message

MMNST is a rare, aggressive tumor with characteristic imaging features due to melanin pigmentation. Accurate diagnosis requires histopathological confirmation, and early surgical intervention is critical for optimal outcomes as the prognosis is poor due to high potential of recurrence and metastasis. Complete resection with or without adjuvant therapy is critical for management.

## Discussion

MMNST is a rare, aggressive neoplasm arising from Schwann cells and characterized by melanocytic differentiation^[^7^]^. MMNTs typically present as well-circumscribed masses of over 5 cm in size^[^8^]^. They are usually encapsulated by a thin fibrous membrane, often occur along the spinal nerve roots, and commonly manifest with progressive pain, frailty, and paresthesia along the corresponding dermatome, often due to nerve root compression^[^9^]^. MMNSTs exhibit no sex predilection^[^9^]^. Extra-dural MMNSTs are located in the epidural space within spinal cord and can infiltrate the spinal cord and invade adjacent vertebrae as well. A significant portion of these tumors (29%) remains asymptomatic, complicating early detection^[^10^]^. In some cases, the tumor may be cutaneous lesions, which may mimic melanomas due to their dark pigmentation. In this case, the tumor had a bluish capsule and invaded the lumbosacral junction involving L5 lower segment, S1 and S2 nerve roots in the epidural space. The tumor was approaching the L5 vertebral body from the below. Microscopy revealed that these tumors exhibit fascicles or nests of neoplastic Schwann cells characterized by the accumulation of melanin pigment in the cytoplasm contributing to its characteristic dark appearance^[^11^]^. Patients with sacral MMNSTs typically present with lower back pain, sciatica-like pain radiating down to one or both legs with weakness and sensory deficit in the lower limbs. In addition, sacral MMNSTs may also lead to sphincter dysfunction. In this case, the patient presented with progressive low-back pain, radiating pain left leg along with tingling sensation. The patient also had decreased perineal sensations, but sphincter functions were intact. The genetics and pathogenesis of the MMNST are complex and not fully understood. The cytogenetic studies have revealed association of recurrent monosomy of chromosome 22q, and variable chromosomal gains and losses on chromosomes 1, 21, and 17p. Trisomy 6p and the presence of ring chromosome 11 are also significant contributors to tumor pathogenesis^[^12^]^. A unique case of involving a missense DDR2 mutation (Q231K) has also been reported, although the precise role of this mutation in pathogenesis of MMNST remains unclear^[^13^]^. MMNSTs co-express both neural crest markers, such as S100/SOX10, alongside with melanocytic markers like HMB45 and Melan-A, reflecting their complex differentiation patterns^[^14^]^.

MMNSTs were previously classified as benign in the 2013 WHO classification but were reclassified as malignant in the 2020 WHO classification due to their aggressive behavior^[^15^]^. Recent studies have reported frequent local recurrence observed in 35% of the cases and metastatic spread observed in 26%–44% of the cases^[^16^]^. MMNST is an exceptionally rare tumor, comprising 1% of all nerve sheath tumors, with fewer than 200 cases reported in the literature^[^8^]^. It is important to note that earlier reports might have classified similar lesions under the umbrella of melanotic schwannomas^[^17^]^, and with recent classifications by the WHO, the number of cases meeting current MMNST criteria remains limited.

Predicting the behavior of MMNST is also challenging^[^18^]^ because MMNST has no strict criteria for malignancy and metastases can occur even in the absence of worrisome histological features such as the presence of large nuclei, prominent macro-nucleoli, and brisk mitotic activity^[^19^]^. Recurrence and metastases have been reported even after two decades of diagnosis. The most common sites include the lung, and pleura, but other locations such as mediastinum, diaphragm, pericardium, endocardium, bone, liver, and spleen may also have metastatic spread^[^10^]^. MRI/CT imaging helps in diagnosis of MMNTs, with MRI being particularly helpful. MMNST exhibits distinctive features on MRI due to the paramagnetic effects of melanin, which influences MRI signals. The tumor typically appears brighter or hyperintense on T1WI and darker or hypointense on T2WI. The presence of hemorrhage may further enhance imaging features of MRI scans^[^20^]^. The MRI scans in our case also demonstrated similar patterns, i.e., hyperintense on T1 and hypointense on T2.

In our case, the initial radiological impression of a pigmented extradural lesion in the lumbosacral spine raised suspicion for multiple possibilities. However, the absence of a primary cutaneous or mucosal melanoma, along with negative systemic findings, helped rule out metastatic melanoma^[^21^]^. Although the tumor exhibited melanin-rich spindle cells, but there was no junctional activity or epithelioid cell morphology typical of melanoma. Immunohistochemistry further guided the diagnosis: diffuse positivity for S-100, and HMB-45 supported melanocytic differentiation, but the lack of cytokeratin and epithelial membrane antigen (EMA) expression excluded carcinoma and chordoma^[^22^]^, respectively. Ultimately, the definitive diagnosis of MMNST was made based on the convergence of radiologic characteristics, intra-operative findings of a pigmented encapsulated mass, and histopathological and immunohistochemistry features^[^23^]^.

Surgical resection is the primary treatment modality for MMNST^[^24^]^. It is crucial to achieve the gross total tumor resection to reduce the risk of recurrence and improve outcomes. Surgical resection of the tumor improves symptoms via decompression of the neurological structures, as in this case, the patient had sensory deficit due to compression of mainly S1 and S2, and lower L5 segment, which were relieved after the removal of tumor. The role of adjuvant radiotherapy remains controversial, especially in cases where there are no clear morphological signs of malignancy^[^25^]^. In such cases, adjuvant treatment is not recommended, and close radiologic and clinical follow-up is advised to monitor the patient. Adjuvant radiotherapy may be considered in the presence of potential indicators for malignancy, incomplete tumor resection, recurrence, or metastases. However, no standard protocols exist up to date for radiotherapy in MMNST. Therefore, the decision to use radiotherapy varies from case to case and is based on tumor’s behavior and patient prognosis. In this case, the patient was referred to oncologist for further adjuvant therapy. However, the patient exhibited no morphological sign of recurrence and his metastatic work-up was also negative. So, the patient required no adjuvant therapy in this case. Given the malignant potential of MMNSTs, a thorough systemic evaluation is imperative. While they may histologically mimic benign lesions, their biological behavior is often deceptive, with reports of early distant spread. Our case emphasizes the importance of a multimodal imaging approach, particularly CECT and bone scans, to rule out occult metastases prior to initiating further oncological management

### Limitations

In this article, we report a single patient’s case, which limits its generalizability. Due to its rarity, there is limited literature available for comparison and validation of the findings. There was also no genetic work-up done in this case.

## Conclusion

MMNST is an exceedingly rare and diagnostically challenging neoplasm with a deceptive histological appearance and a potentially aggressive clinical course. This case highlights the critical importance of maintaining a broad differential diagnosis for spinal tumors and relying on definitive histopathological and immunohistochemical analysis for accurate diagnosis. The patient’s favorable outcome following surgical decompression, negative metastatic work-up, and 1-year disease-free follow-up highlights the value of early detection and timely neurosurgical intervention. This case report adds to the limited existing literature and highlights the need for multidisciplinary evaluation and individualized oncologic planning in managing such rare tumors.

## References

[1] Hammad RM. Malignant melanotic nerve sheath tumors: a review of clinicopathologic and molecular characteristics. J Microsc Ultrastruct 2023;11:125. 38025185 10.4103/jmau.jmau_5_22 PMC10679827

[2] Ghaith AK Johnson SE El-Hajj VG. Surgical management of malignant melanotic nerve sheath tumors: an institutional experience and systematic review of the literature. 2023. Cited 10 October 2024. https://thejns.org/spine/view/journals/j-neurosurg-spine/40/1/article-p28.xml 10.3171/2023.8.SPINE23427 37862711

[3] Benson JC Marais MD Flanigan PM. Malignant melanotic nerve sheath tumor. Am J Neuroradiol 2022;43:1696–99. 36302602 10.3174/ajnr.A7691

[4] Yeom JA Song YS Lee IS. Malignant melanotic nerve sheath tumors in the spinal canal of psammomatous and non-psammomatous type: two case reports. World J Clin Cases 2022;10:8735–41. 36157803 10.12998/wjcc.v10.i24.8735 PMC9453363

[5] Li Z Niu Y. Malignant melanotic nerve sheath tumor of the parotid gland: a case report and literature review. Ear Nose Throat J. 2023;1455613221145803. 10.1177/01455613221145803 36597949

[6] Sohrabi C Mathew G Maria N. The SCARE 2023 guideline: updating consensus Surgical CAse REport (SCARE) guidelines. Int J Surg 2023;109:1136. 37013953 10.1097/JS9.0000000000000373 PMC10389401

[7] Franca RA Di Crescenzo RM Ugga L. The “pigmented side” of nerve sheaths: malignant melanotic nerve sheath tumor. Int J Surg Pathol 2024:10668969241295689. 10.1177/10668969241295689 39563513

[8] Lin KY Chen L Hung SW. A para-aortic malignant melanotic nerve sheath tumor mimicking a gastrointestinal stromal tumor: a rare case report and review of literature. BMC Surg 2022;22:293. 35902891 10.1186/s12893-022-01727-4 PMC9331146

[9] Bonomo G Gans A Mazzapicchi E. Sporadic spinal psammomatous malignant melanotic nerve sheath tumor: a case report and literature review. Front Oncol 2023;13:1100532. 36910634 10.3389/fonc.2023.1100532 PMC9998981

[10] Alexiev BA Chou PM Jennings LJ. Pathology of melanotic schwannoma. Arch Pathol Lab Med 2018;142:1517–23. 29372846 10.5858/arpa.2017-0162-RA

[11] Keskin E Ekmekci S Oztekin O. Melanotic schwannomas are rarely seen pigmented tumors with unpredictable prognosis and challenging diagnosis. Case Rep Pathol 2017;2017:1807879. 29109888 10.1155/2017/1807879 PMC5646290

[12] Wang L Zehir A Sadowska J. Consistent copy number changes and recurrent PRKAR1A mutations distinguish melanotic schwannomas from melanomas: SNP-array and next generation sequencing analysis. Genes Chromosomes Cancer 2015;54:463–71. 26031761 10.1002/gcc.22254 PMC6446921

[13] Roman Hernandez EM Valasareddi SL Adkison J. Can discord domain-containing receptor 2 mutation act as a disease modifier for PRKAR1A associated melanotic schwannoma? Case Rep Oncol 2021;14:826–31. 34248546 10.1159/000515331 PMC8255715

[14] Torres-Mora J Dry S Li X. Malignant melanotic schwannian tumor: a clinicopathologic, immunohistochemical, and gene expression profiling study of 40 cases, with a proposal for the reclassification of “melanotic schwannoma”. Am J Surg Pathol 2014;38:94–105. 24145644 10.1097/PAS.0b013e3182a0a150

[15] Sbaraglia M Bellan E Dei Tos AP. The 2020 WHO classification of soft tissue tumours: news and perspectives. Pathologica 2020;113:70–84. 33179614 10.32074/1591-951X-213 PMC8167394

[16] Buckley B Delaney F Aird JJ. Pararenal malignant melanotic nerve sheath tumour: a rare tumour in an unfamiliar location. BMJ Case Rep 2022;15:e252107. 10.1136/bcr-2022-252107 PMC9379493 35961688

[17] Xiang Z Feng M Gao Q. Malignant thoracic intraspinal melanotic schwannoma. Asian J Surg 2023;46:4794–95. 37290982 10.1016/j.asjsur.2023.05.096

[18] Shui C Davey L Scholsem M. Leptomeningeal dissemination of a malignant melanotic nerve sheath tumor: a case report and review of the literature. Surg Neurol Int 2022;13:59. 35242425 10.25259/SNI_31_2022 PMC8888312

[19] American Journal of Neuroradiology. Malignant Melanotic Nerve Sheath Tumor (MMNST). Cited 7 April 2025. https://www.ajnr.org/ajnr-case-collections-diagnosis/malignant-melanotic-nerve-sheath-tumor-mmnst?__cf_chl_tk=r_vontNSbADPo1o7hGv99KbNNqLyfA2g2amraVNR3jU-1744034519-1.0.1.1-iU556n1swDS5zhFVc6x_hgvN3lwB.vv.wmrRfNX0Plw

[20] Siddiqui FM Bekker SV Qureshi AI. Neuroimaging of hemorrhage and vascular defects. Neurother J Am Soc Exp Neurother 2011;8:28. 10.1007/s13311-010-0009-x PMC3075731 21274683

[21] Zhang Q Mao Z Huang D. Pelvic metastatic melanoma mimicking malignant schwannoma: a case report. Asian J Surg 2023;46:5993–94. 37739893 10.1016/j.asjsur.2023.09.036

[22] Andrei V Haefliger S Baumhoer D. Superficial mesenchymal tumours expressing epithelial markers on immunohistochemistry: diagnostic clues and pitfalls. Semin Diagn Pathol 2023;40:238–45. 37147159 10.1053/j.semdp.2023.04.016

[23] Belakhoua SM Rodriguez FJ. Diagnostic pathology of tumors of peripheral nerve. Neurosurgery 2021;88:443–56. 33588442 10.1093/neuros/nyab021 PMC7884141

[24] Seres R Hameed H McCabe MG. The multimodality management of malignant peripheral nerve sheath tumours. Cancers (Basel) 2024;16:3266. 39409887 10.3390/cancers16193266 PMC11475700

[25] Collart J Vandeponseele M Bosschaert P. Intracranial melanotic schwannomas: rare and distinctive tumors to know due to their risk of recurrence and metastases. J Belg Soc Radiol 2018;102:15. 30039029 10.5334/jbsr.1359 PMC6032525

